# The complete chloroplast genome of *Coreopsis lanceolata* L. and its phylogenetic analysis

**DOI:** 10.1080/23802359.2025.2555460

**Published:** 2025-09-02

**Authors:** Chunfeng Wan, Dexin Wang, Xiaoqin Zhang, Hongling Liang, Junwei Zhang

**Affiliations:** a College of Agriculture and Bioengineering (Peony College), Heze University, Heze, P. R. China; b Heze Municipal Bureau of Agriculture and Rural Affairs, Heze, P. R. China

**Keywords:** Coreopsis lanceolata, Asteraceae, chloroplast genome, phylogenetic analysis

## Abstract

*Coreopsis lanceolata* L. 1753 is a perennial herb of the family Asteraceae, often cultivated as an ornamental flower. The species has also been reported to contain a wide range of phytochemicals and to exhibit diverse pharmacological activities. To better understand the genetic information of *C. lanceolata*, its complete chloroplast genome was assembled and characterized for the first time in this study. The chloroplast genome was 150,448 bp long and contained 129 genes, including 86 protein-coding genes, 35 tRNA genes, and eight rRNA genes. A rearrangement involving double inversions was observed in the chloroplast genome when compared with that of *Barnadesia caryophylla*. Phylogenomic analysis indicated that *C. lanceolata*, *Bidens frondosa*, *B. tripartita*, and *B. pilosa* formed a monophyletic clade. The findings of this study provide support for future research on the evolution and potential pharmaceutical applications of this species.

## Introduction

*Coreopsis lanceolata* L. 1753 (Asteraceae), commonly known as lance-leaf coreopsis, is a perennial herb native to North America but widely cultivated in Asia and Europe for its ornamental, ecological, and medicinal value (Kim et al. 2020; Zhu et al. 2023). This species is notable for its drought tolerance, adaptability to diverse soils, bright yellow flowers (4–6 cm diameter) with toothed margins, and extended flowering season (spring to summer). These features make it valuable for urban landscaping and pollinator conservation (Kim et al. 2020, 2021).

Beyond its ornamental appeal, *C. lanceolata* has been extensively studied for its phytochemical diversity and pharmacological properties. Modern studies have identified several bioactive compounds in its flowers and leaves, including flavonoids, chalcones, aurones, and polyacetylene glycosides (Kim et al. 2020, 2021). Among these, flavonoids such as leptosidin, leptosin, isoquercetin, and astragalin exhibit strong antioxidant and anti-inflammatory effects (Kim et al. 2021).

In phylogenetic studies of *C. lanceolata*, Sherff et al. (1955) noted that the boundaries between the genera *Bidens*, *Coreopsis*, and *Cosmos* are indistinct. Banfi et al. (2017) supported merging the three genera into a single genus, *Bidens*, a proposal reinforced by ITS sequence analysis (Kimball and Crawford 2004). However, Hind et al. (2023) criticized this approach for its heavy reliance on a single molecular marker and insufficient consideration of morphological stability in long-cultivated horticultural varieties such as *C. lanceolata*. Some taxonomists, including Strother and Flora of North America Editorial Committee (2006), still retain *Coreopsis* a separate genus.

The chloroplast (cp) genome is critical for understanding evolutionary relationships, genetic diversity, and the potential for metabolic engineering in medicinal plants (Jansen et al. 2005). However, the cp genome of *C. lanceolata* has not yet been investigated. This study aimed to assemble and characterize the complete chloroplast genome of *C. lanceolata*, thereby providing valuable insights for future studies on its evolution and pharmaceutical development.

## Materials and methods

**Figure 1.**
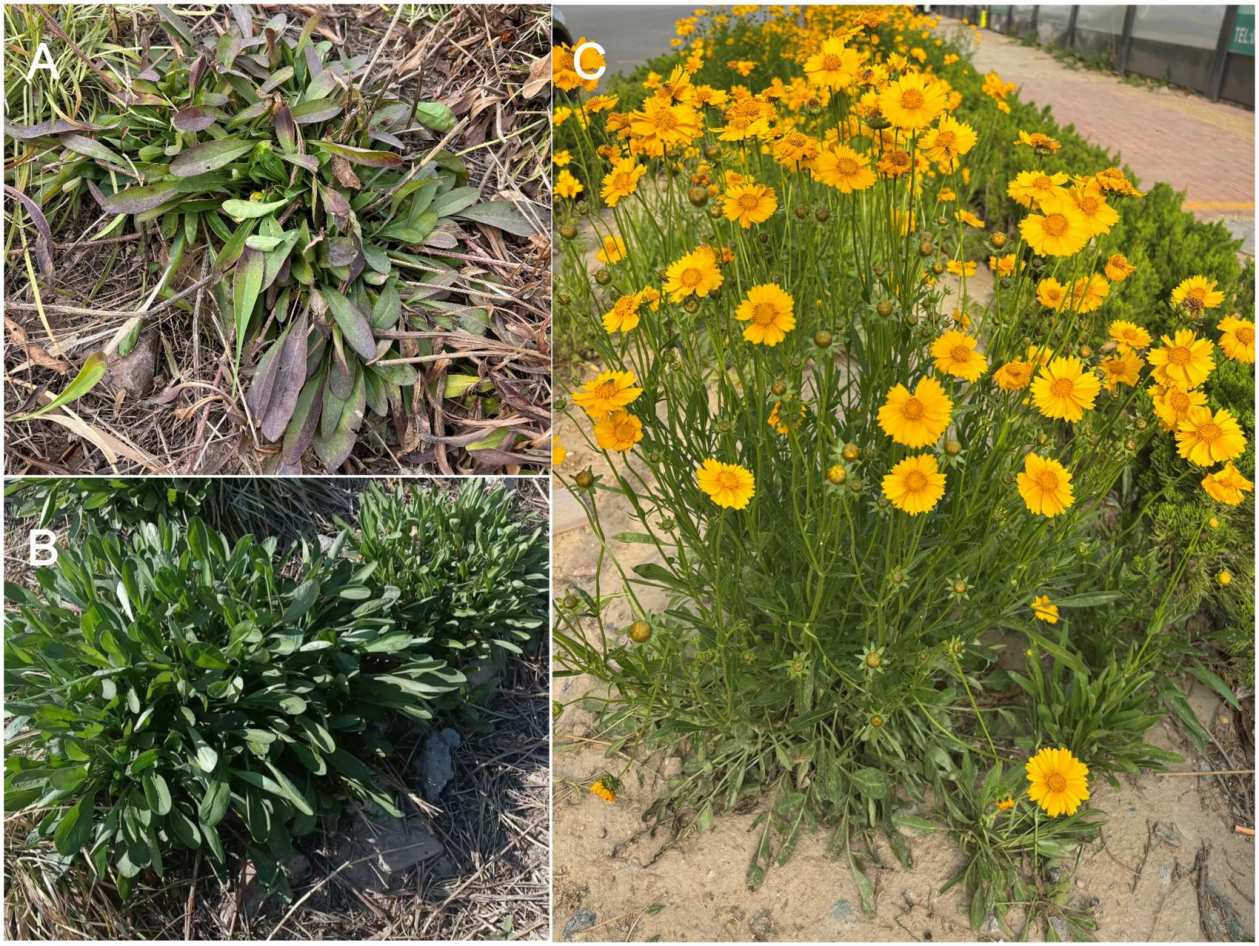
Field photograph of *Coreopsis lanceolata*. Location: 35°16′55.37″N, 115°27′38.77″E. Photographer: Chunfeng Wan. (A) *C. lanceolata* during overwintering, (B) after overwintering, and (C) at flowering stage. Key morphological features: perennial herb (30–70 cm tall) with spindle-shaped roots; basal leaves opposite, spatulate to linear-oblanceolate; stem leaves linear; solitary yellow capitula; fruits orbicular to elliptical; flowering from May to September.

The fresh leaves of *C. lanceolata* used for sequencing were obtained from Peony District, Heze City, Shandong Province, China (35°16′55.37″N, 115°27′38.77″E) (Figure 1). A specimen was deposited in the Heze University Herbarium (contact: Chunfeng Wan, wancfeng@126.com) under the accession number HZ20250102. The identification and sample collection were performed by Chunfeng Wan.

Whole genomic DNA was extracted using the Plant Genomic DNA Kit (Tiangen Biotech, Beijing, China). The DNA was sheared into ∼300 bp fragments for library construction, followed by 150 bp paired-end sequencing on the Illumina NovaSeq 6000 platform (Benagen Tech, Wuhan, China). Trimmomatic (v0.35) (Bolger et al. 2014) with default parameters was used to filter raw reads by removing adapters and low-quality reads. Approximately 28 GB of clean reads (fastq format) were assembled using GetOrganelle (v1.7.1) (Jin et al. 2020). The complete chloroplast genome was annotated using the online tool CPGAVAS2 (Shi et al. 2019) and manually refined in Apollo (Pontius 2018). The annotated genome was submitted to the GenBank under accession number PV694530. The circular genome map was generated using CPGview (Liu et al. 2023).

To investigate the phylogenetic position of *C. lanceolata*, 11 chloroplast genomes from other Coreopsideae species of Asteraceae were downloaded from the GenBank. *Larrea tridentata* and *Tribulus terrestris* were used as outgroups. Whole chloroplast genome sequences were aligned with MAFFT software with default parameters (Katoh and Standley 2016), and a maximum-likelihood (ML) phylogenetic tree was constructed using IQ-TREE (v2.0) (Nguyen et al. 2015) under the best-fit model TVM+F + G4 with 1000 bootstrap replicates.

## Results

**Figure 2.**
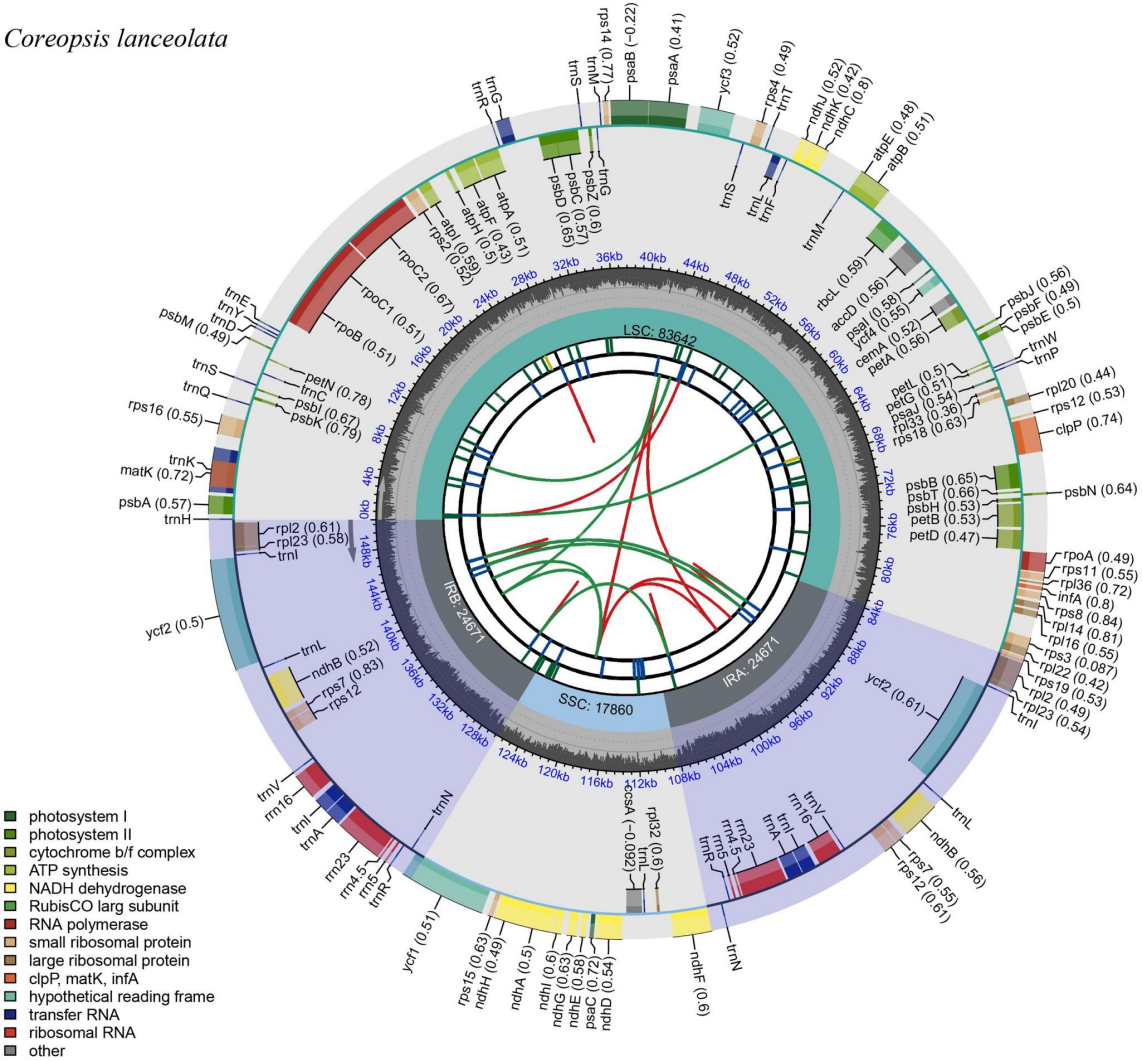
The complete chloroplast genome of *Coreopsis lanceolata*. The map contains six tracks. From the center outward, the first track displays the dispersed repeats. The second track shows long tandem repeats as short blue bars. The third track shows short tandem repeats or microsatellite sequences as short bars with different colors. The small single-copy (SSC), inverted repeat (IRA and IRB), and large single-copy (LSC) regions are shown on the fourth track. The GC content along the genome is plotted on the fifth track. The genes are shown on the sixth track. Genes belonging to different functional groups are color-coded. Genes on the inside and outside of the map are transcribed in clockwise and counterclockwise directions, respectively. For protein-coding genes, letters after names indicate subunits or family members; numbers denote variants. For rRNA genes, numbers represent rRNA size in Svedberg units. For tRNA genes, letters indicate the recognized amino acid. For unknown-function genes, numbers refer to hypothetical coding genes.

The chloroplast genome sequence of *C. lanceolata* is 150,844 bp length and displays a typical quadripartite structure. The assembled genome achieved an average sequencing depth of 3249.63 ×, with a minimum depth of 472 × and a maximum depth of 4311 × (Supplemental Figure S1). The genome consists of two IR regions of 24,671 bp each, separated by a largesingle-copy (LSC) region of 83,642 bp and a small single-copy (SSC) region of 17,860 bp (Figure 2). When compared with the chloroplast genome (OM892817.1) of *Barnadesia caryophylla* (Barnadesioideae, Asteraceae), a structural rearrangement was defined in the LSC region, consisting of a large ∼18.8 kb inversion containing a smaller nested ∼2.7 kb inversion (Supplemental Figure S2). This rearranged region includes 16 genes: *trn*C, *pet*N, *psb*M, *trn*D, *trn*Y, *trn*E, *rpo*B, *rpo*C1, *rpo*C2, *rps*2, *atp*I, *atp*H, *atp*F, *atp*A, *trn*R, and *trn*G.

The chloroplast genome exhibits a variable GC content, with an overall value of 37.5%. The IR regions have the highest GC content (43.1%), followed by the LSC (35.6%) and SSC (31.2%) regions. A total of 129 genes were predicted, including 86 protein-coding genes (PCGs), eight rRNA genes, and 35 tRNA genes. Six unique PCGs (*rps*12, *rpl*23, *ycf*2, *ndh*B, *rps*7, and *rps*12), seven unique tRNA genes (*trn*I, *trn*L, *trn*V, *trn*A, *trn*R, and *trn*N), and four unique rRNA genes (*rrn*16S, *rrn*23S, *rrn*4.5S, and *rrn*5S) were located in the IR regions. Within the entire chloroplast genome, nine PCGs (*atp*F, *ndh*A, *ndh*B × 2, *pet*B, *pet*D, *rpl*16, *rpl*2, and *rps*16) each contained one intron, while two PCGs (*ycf*3 and *clp*P) contained two introns. Additionally, seven tRNA genes (*trn*K, *trn*S, *trn*L-UAA, *trn*E × 2, and *trn*A × 2) contained one intron. The *rps*12 gene was identified as a trans-splicing gene (Supplemental Figure S3), while the structures of cis-splicing PCG genes were presented in Supplemental Figure S4.

**Figure 3.**
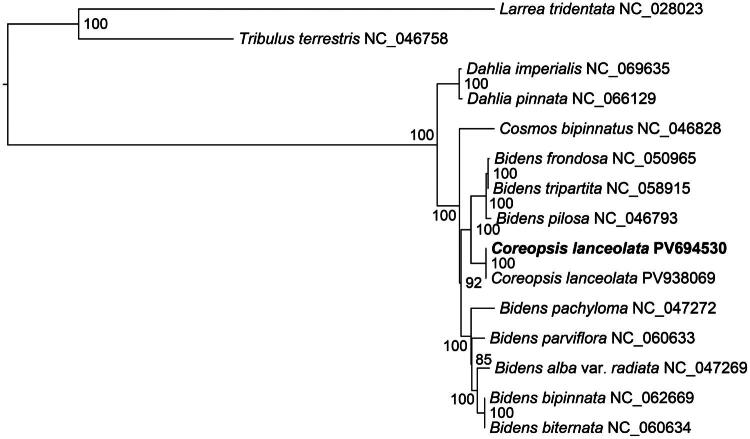
Maximum-likelihood phylogeny of *Coreopsis lanceolata* and its close relatives. The bootstrap values based on 1000 replicates were shown on each node in the phylogenetic tree. The 14 species were downloaded from the GenBank: *Dahlia imperialis* (NC_069635), *D. pinnata* (NC_066129) (Xue et al. 2019), *Cosmos bipinnatus* (NC_046828) (Jiang et al. 2019), *Bidens frondosa* (NC_050965) (Li et al. 2020), *B. tripartite* (NC_058915) (Wu et al. 2022), *B. pilosa* (NC_046793) (Lin et al. 2020), *C. lanceolata* (PV694530, the new reported chloroplast genome labeled by bold font), *B. pachyloma* (NC_047272) (Knope et al. 2020), *B. parviflora* (NC_060633) (Wu et al. 2022), *B. alba* var. *radiata* (NC_047269) (Knope et al. 2020), *B. bipinnata* (NC_062669) (Zhang et al. 2023), *B. biternate* (NC_060634) (Wu et al. 2022), *Tribulus terrestris* (NC_046758, outgroup) (Yan et al. 2019) and *Larrea tridentata* (NC_028023, outgroup) (Wang et al. 2022).

The phylogenetic analysis revealed that the two *C. lanceolata* accessions formed a monophyletic group with *Bidens frondosa*, *B. tripartita*, and *B. pilosa* (Figure 3). This clade received maximum bootstrap support (100%), confirming their close evolutionary relationship. Additionally, *Dahlia* and *Cosmos* species formed separate monophyletic groups, while *Cosmos*, *Bidens*, and *C. lanceolata* together constituted a broader monophyletic clade. These results suggest that *C. lanceolata* is more closely related to *Bidens* and *Cosmos* species, although further sampling of additional *Coreopsis* species will be necessary to validate their phylogenetic relationship.

## Conclusions and discussion

This study reports, for the first time, the complete chloroplast genome of *C. lanceolata*. The genome measures 150,844 bp and contains 129 predicted genes, exhibiting a typical quadripartite structure. Structure analysis revealed rearrangements in the LSC region, consisting of two nested inversions. The double inversion detected in *C. lanceolata* appears to be conserved across most major clades of Asteraceae, with the exception of Barnadesioideae (Pascual-Díaz et al. 2021).

Our phylogenetic analysis demonstrated that *C. lanceolata* formed a strongly supported monophyletic group with *Bidens frondosa*, *B. tripartita*, and *B. pilosa* within the Coreopsideae of Asteraceae. These chloroplast genome-based results partially agree with Banfi et al. (2017), who proposed the transfer of some *Coreopsis* species to the genus *Bidens* within the tribe Coreopsideae (Asteraceae). At the same time, our findings highlight the importance of the integrating molecular and morphological evidence. While Hind et al. (2023) emphasized differences between *C. lanceolata* and *Bidens* in terms of the shape of the achene wing, which are indeed key criteria for traditional classification. But between *C. lanceolata* and *Bidens*, molecular systematics reveals can uncover evolutionary histories that traditional morphology may overlook, such as obscured by convergent evolution or the loss of ancestral traits or convergent evolution. The chloroplast genome data presented here suggest that the genetic similarity between *C. lanceolata* and *Bidens* species may reflect shared ancestry rather than superficial morphological divergence. These findings provide a genomic framework for future studies on the evolutionary history of Coreopsideae and potential pharmaceutical applications of *C. lanceolata*.

## Supplementary Material

Supplemental Material

## Data Availability

The complete chloroplast genome sequence of *Coreopsis lanceolata* in this study has been submitted to the NCBI database under the accession number PV694530. https://www.ncbi.nlm.nih.gov. The associated BioProject, BioSample, and SRA numbers are PRJNA1146888, SAMN48785330, and SRR33748099.
