# Supplementary material for: The complete chloroplast genome of Coreopsis lanceolata L. and its phylogenetic analysis
Source: Mitochondrial DNA B Resour. 2025 Sep 2;10(10):899–903. doi: 10.1080/23802359.2025.2555460 (PMC12406329; doi:10.1080/23802359.2025.2555460)
Supplement: Supplemental Material [file TMDN_A_2555460_SM9666.docx]

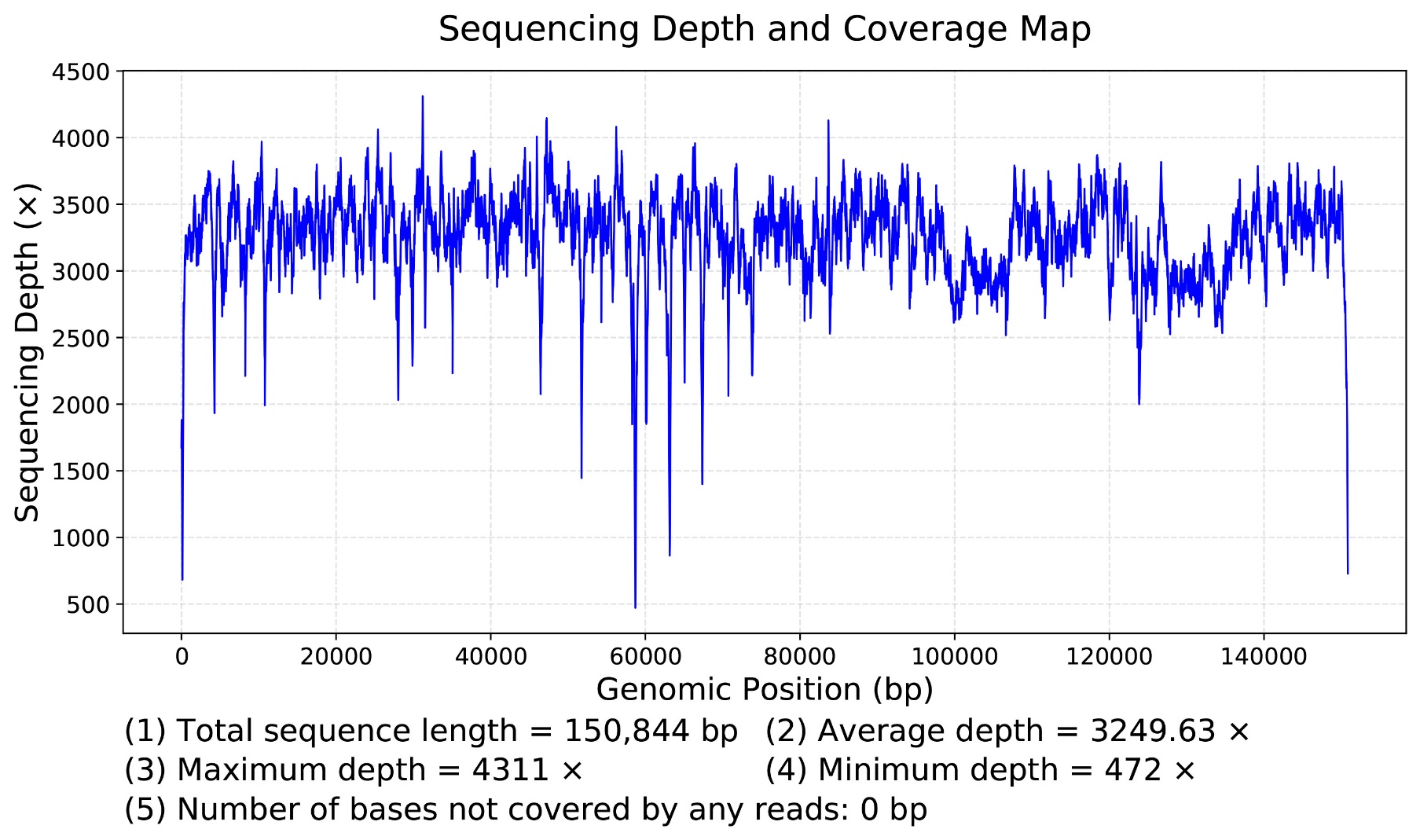


**Figure S1. The coverage depth of the chloroplast genome assembly *Coreopsis lanceolata.***


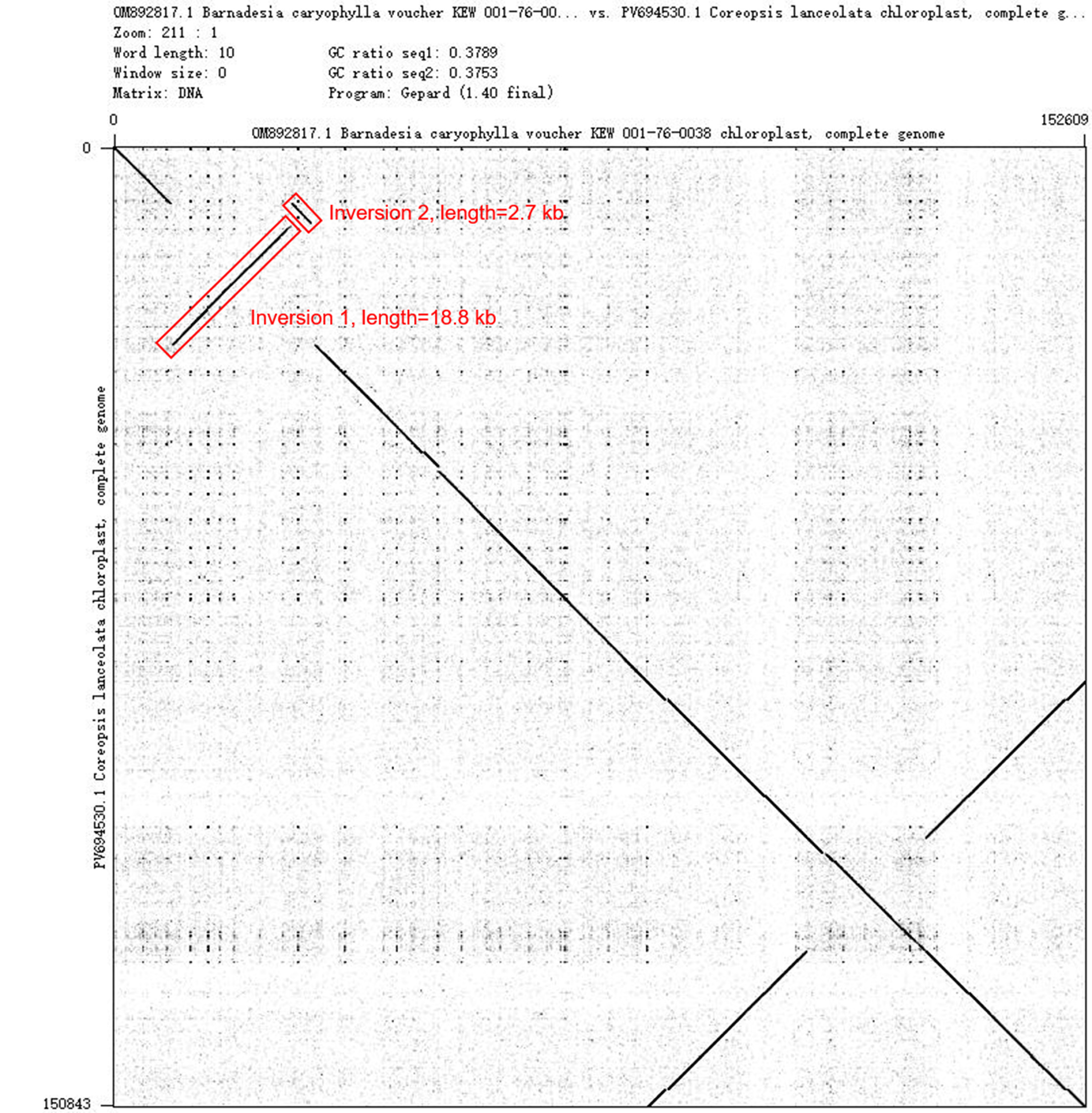


**Figure S2. Dotplot map between the chloroplast genomes of *Barnadesia caryophylla* (OM892817, X-axis) and *Coreopsis lanceolata* (PV694530, Y-axis).** A rearrangement event in the LSC consisting of a double inversion: one large inversion of ~18.8 kb and one small inversion of ~2.7 kb nested within the large one.


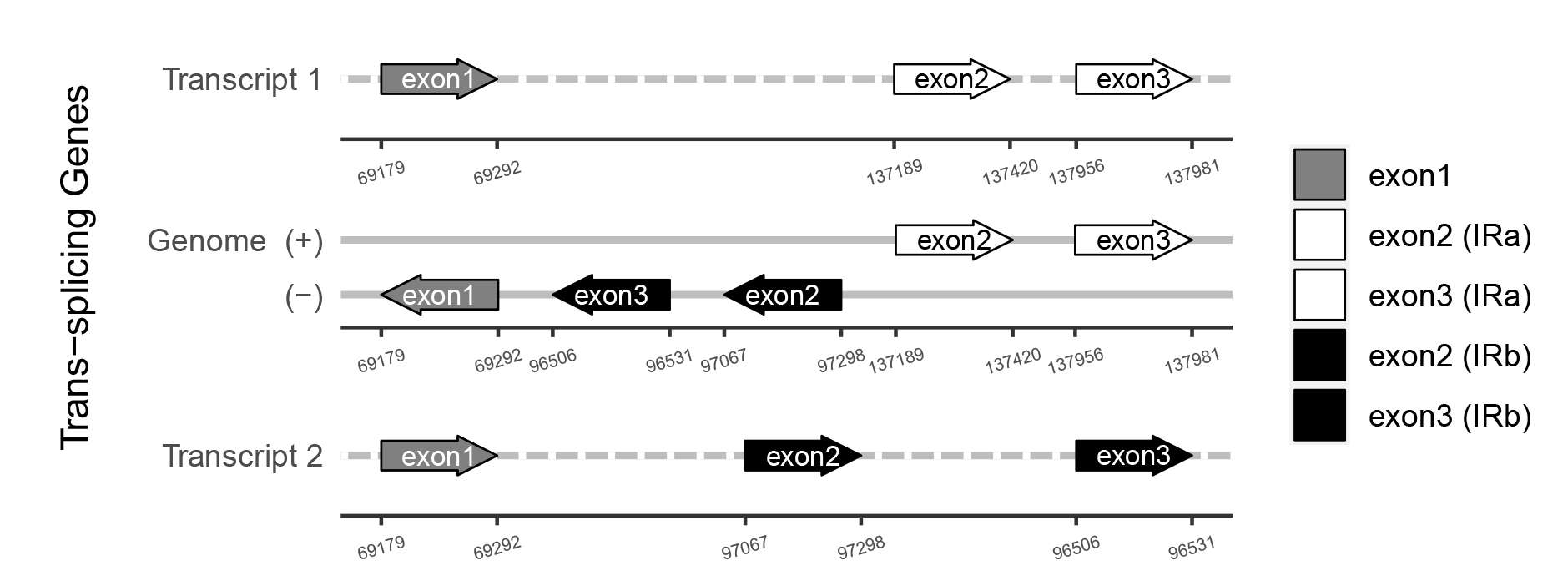


**Figure S3. Schematic map of the trans-splicing gene *rps*12 in the chloroplast genome of *Coreopsis lanceolata*.**


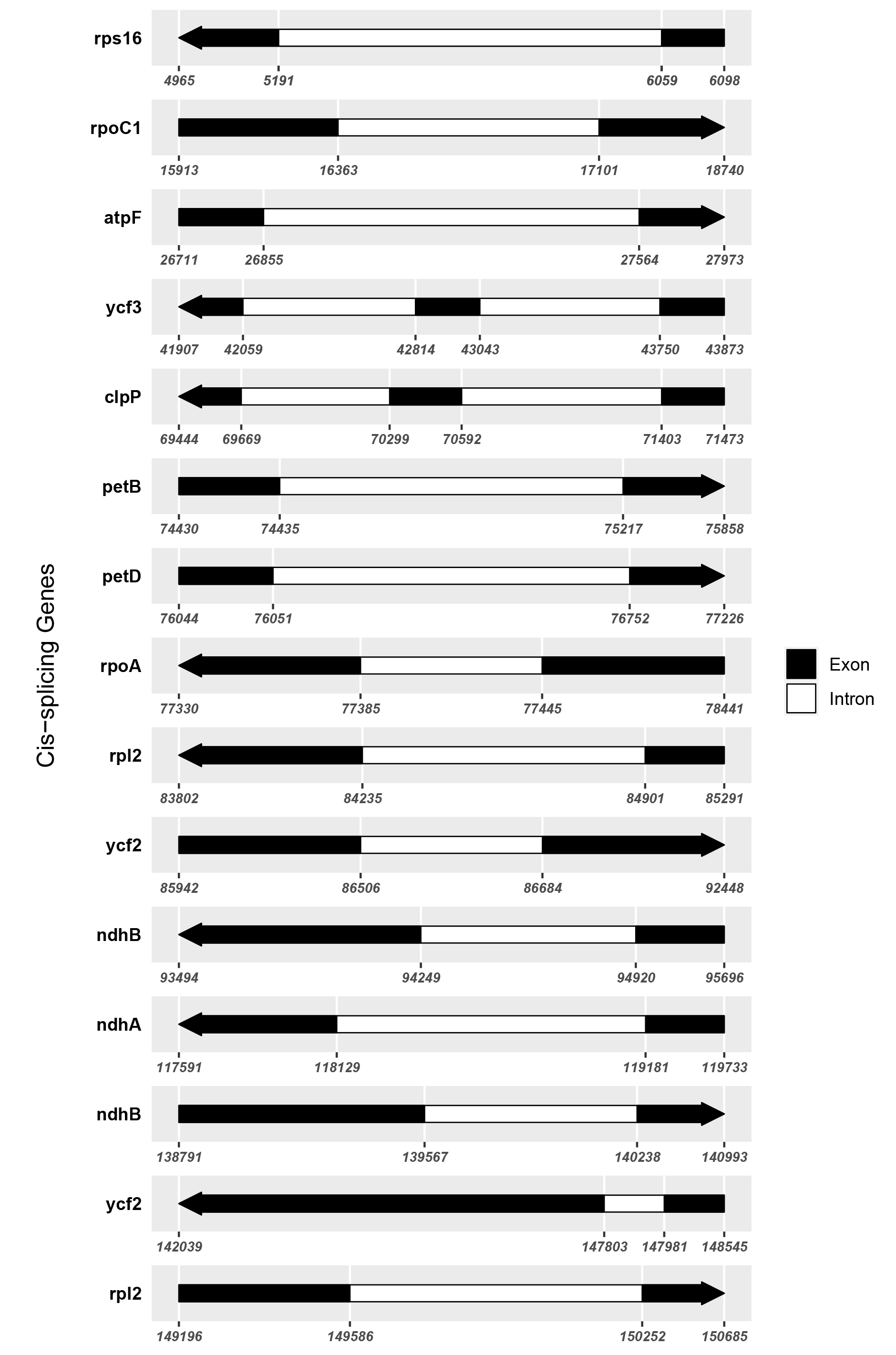


**Figure S4. Schematic map of the cis-splicing genes in the chloroplast genome of *Coreopsis lanceolata*.** Exons and introns are shown in black and white, respectively. The arrow indicates the sense direction of the gene.
